# Median Nerve Cavernous Hemangioma

**DOI:** 10.18869/nirp.bcn.8.3.255

**Published:** 2017

**Authors:** Mohammed Al-Garnawee, Marwan Najjar

**Affiliations:** 1. Department of Surgery, Faculty of Medicine, American University of Beirut, Beirut, Lebanon.

**Keywords:** Median nerve, Cavernous hemangioma, Peripheral nerve lesions, Entrapment syndromes

## Abstract

Hemangiomas of the median nerve are extremely rare; only 12 cases have been reported in the literature. We discuss a patient who presented with paresthesia and pain along the distribution of the left median nerve secondary to a cavernoma of the proximal part of the nerve as suspected on MRI scan. Total removal of the mass was achieved with immediate relief of the symptoms and no neurologic deficit. We conclude that despite being quite rare, the diagnosis of occult vascular lesions of peripheral nerves such as the median nerve, should be considered, especially when other common pathologies are excluded.

## 1. Introduction

Peripheral nerve tumors are rare. They can be classified according to their origin as either nerve sheath tumors or non-neural sheath tumors (Kim, Murovic, Tiel, Moes, & Kline, 2005). The most frequent nerve sheath tumors are benign and include schwannomas and neurofibromas (Schroder, 2001). Benign non-neural sheath tumors mainly include lipomas and vascular tumors. Malignant peripheral nerve sheath tumors are much less frequent and could include metastasis. Very few cases of vascular lesions involving the peripheral nervous system have been reported. In this paper, we report on a hemangioma involving the sheath of the left median nerve.

## 2. Case Report

**Figure 1.**
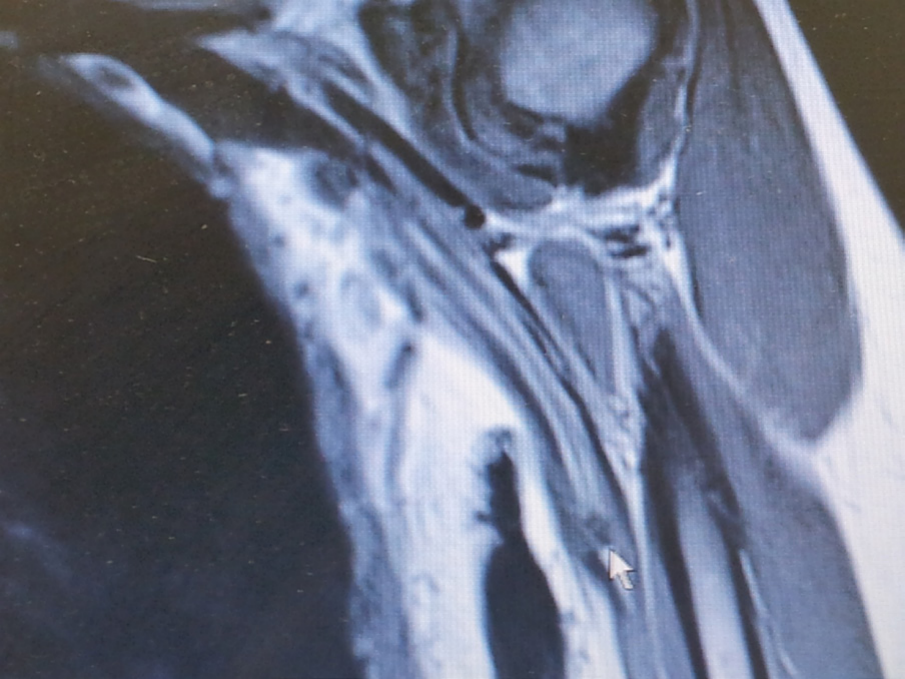
Magnetic resonance imaging of the median nerve showing a hypointense lesion of the nerve at the level of the midarm (arrow).

A 43-year-old female presented with 18 months history of left upper extremity paresthesia and pain along the median nerve distribution, which got progressively more severe over time, though with no motor weakness. The patient failed medical treatment. EMG and nerve conduction testing were normal. Magnetic resonance imaging (MRI) results of the cervical spine were within normal limits. MRI of the left upper extremity showed a 9-mm mass arising from the left median nerve suggestive of a cavernous hemangioma (Figure 1).

**Figure 2.**
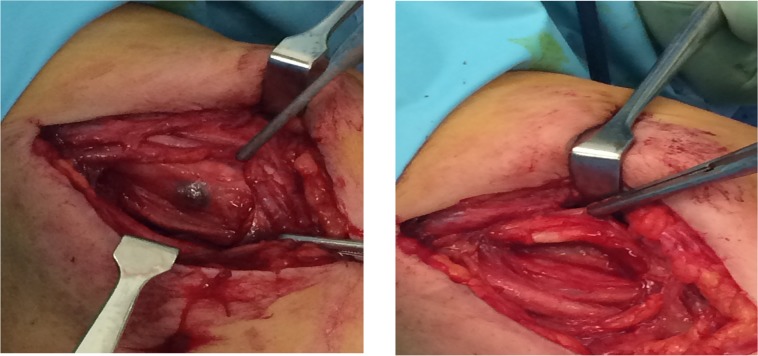
Intraoperative picture after exposure of the median nerve. a: A small black lesion is seen involving the median nerve. b: Complete microsurgical removal of the lesion.

**Figure 3.**
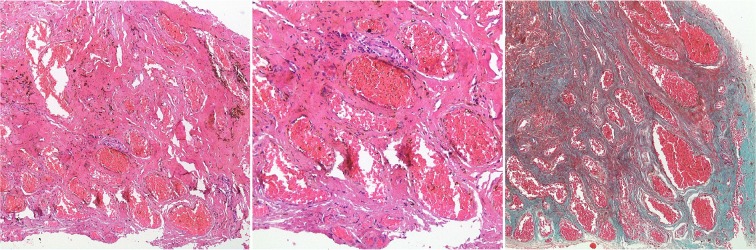
Peripheral (median) nerve epineural hemangioma characterized by dilated venous channels with intervening fibroconnective tissue (Left and middle panel H&E, 100× & 200×; Right panel Trichrome Stain, 100×).

On operation, we managed to identify a small firm black-looking mass closely related to the sheath of the left median nerve in the upper arm (Figure 2a). After careful dissection, and under microscopic magnification, a complete resection of the mass was achieved (Figure 2b). Postoperatively, the patient reported instant relief of her symptoms with no neurological deficit. Histopathological examination showed dilated venous channels with intervening fibroconnective tissue suggestive of cavernous hemangioma (Figure 3). Upon 6 months of follow up, the patient remained asymptomatic without signs of recurrence on MRI.

## 3. Discussion

Benign vascular tumors originating from peripheral nerves are rare. The median nerve is most commonly affected, followed by the tibial, ulnar, digital, sciatic, and superficial peroneal nerves (Chatillon, Guiot, & Jacques, 2007). Only 12 cases of median nerve hemangiomas are reported in the literature (Table 1) (Coessens, De Mey, Lacotte, & Vandenbroeck, 1991; Dogramaci, Kalaci, Sevinç, & Yanat, 2014; Kojima, Ide, Marumo, Ishikawa, & Yamashita, 1976; Louis & Fortin, 1992; Oztekin & Karaarslan, 2003; Patel, Tsai, & Kleinert, 1986; Peled, Iosipovich, Rousso, & Wexler, 1980; Petrovici, 1980; Prosser & Burke, 1987; Sato, 1913; Vekris et al., 2008).

**Table 1.** Median nerve cavernous hemangiomas reported in the literature.

**Outcome**	**Procedure**	**Location**	**Age/Gender**	**Author**
No recurrence, asymptomatic at 6 months follow up	Intra-fascicular dissection and resection of tumor	Carpal tunnel	12/M	Coessens et al. (1991)
No recurrence in two years follow up	Resection	Carpal tunnel	14/F	Dogramaci et al. (2008)
Neurologically normal at six weeks after surgery	Non-specified type of resection	Carpal tunnel	19/F	Kojima et al. (1976)
Asymptomatic for six months follow up	Resection	Proximal 1/3 of the forearm	21/NA	Louis et al. (1992)
No recurrence in six months	Resection	Carpal tunnel	35/F	Oztekin et al. (2003)
Remained symptomatic	Multiple excisions	Carpal tunnel	4/F	Patel et al. (1986)
No recurrence	Recurrence at 2 years after intraneural dissection/patient then had resection with sural nerve graft	Carpal tunnel	15/F	Patel et al. (1986)
Hyperesthesia improved after three weeks and weakness which improved gradually	Partial excision, followed by en bloc resection after 3 years	Carpal tunnel	16/F	Peled et al. (1980)
No recurrence in one year	Resection	Carpal tunnel	22/F	Petrovici. (1980)
Fourth Recurrence treated conservatively Decrease sensation in thumb and index	Multiple excisions	Carpal tunnel	13/F	Prosser et al. (1987)
Ulnar parasthesia	Resection of tumor with involved nerve segment	Carpal tunnel	64/M	Sato. (1913)
No recurrence in three years follow up	Resection	Carpal tunnel	10/F	Vekris et al. (2008)

According to the nerve structure involved, these tumors can be classified into 3 types. Type I is an intraneural extrafascicular malformation that is relatively easily removed with magnification. Type II is an intrafascicular encompassing type that is deemed unresectable because of the possible loss of nerve function secondary to the required dissection. Type III has both intraneural and extraneural components (Louis & Fortin, 1992) (Table 2).

**Table 2.** Classification of peripheral nerve vascular malformations.

**Types**	**Extent of Involvement**
I	Intraneural extrafascicular malformation
II	Intrafascicular
III	Intraneural and extraneural

In the majority of cases, the patients report a palpable mass along the path of the median nerve. Carpal tunnel like syndrome is the most common finding and one case had Raynaud’s phenomenon as an associated presenting symptom (Prosser & Burke, 1987). The differential diagnosis of such lesions includes lipoma, lipofibroma, hamartoma, and intraneural schwannoma (Chatillon et al., 2007).

The diagnostic work up of such lesions includes ultrasonography, nerve conduction studies, and MRI. Regarding sonography, most peripheral nerve sheath tumors share the common features of being hypoechoic and homogeneous, with posterior acoustic enhancement and peripheral nerve continuity, where the finding of peripheral nerve continuity indicates peripheral nerve sheath tumor as the cause (Reynolds et al., 2004).

On MRI, magnetic resonance characteristics reported in the literature include hyperintense signal on T1- and T2- weighted images with fat suppression sequences. These lesions are also noted to enhance after gadolinium administration (Dogramaci et al., 2014). Magnetic Resonance Imaging (MRI) gives useful information regarding the anatomic location, size, and relationship of intraneural hemangioma of the median nerve to surrounding structures and may help differentiate between various tumor types (Ergin, Druckmiller, & Cohen, 1998).

Treatment of such lesions with a conservative approach usually fails and surgery is the treatment of choice (Dogramaci et al., 2014). Total resection of intraneural hemangiomas is curative when possible, whereas partial resection may relieve symptoms. Recurrence, however, may occur which may require en bloc nerve resection and repair with nerve graft (Patel et al., 1986). Cases in which the hemangioma is intraneural but essentially extrafascicular tend to do well with local excision alone (Louis & Fortin, 1992). Most reported peripheral nerve hemangiomas are of the cavernous type although the capillary subtype has been identified. The consensus as to the histogenesis of peripheral nerve hemangioma favors the origin to be in the capillary bed of the epineurium with subsequent extension to the nerve trunk (Schroder, 2001).

Our case, like most of the previously reported cases, is a female with similar presenting symptoms and the same histopathological type (cavernous hemangioma). However, she is older than all the cases except one case. Unlike the majority of cases where the lesions were situated near the carpal tunnel or the palm, our case is the first case of cavernous hemangioma involving the median nerve in a proximal location in the arm.

## 4. Conclusion

In conclusion, despite the rarity of such lesions, cavernous hemangioma should be considered in the differential diagnosis of median nerve lesions, especially in young females with unexplained pain, paresthesia, and a palpable lesion. The diagnosis of such lesions involves a thorough history and physical exam as well as appropriate imaging modalities, especially ultrasonography and MRI. Careful intraoperative dissection of such lesions is important for preserving nerve function and usually results in excellent outcome, whereas en bloc resection with grafting should be reserved for complicated or recurrent cases.
